# Effect of Photosynthetic Photon Flux Density on Paprika Seedling Growth Using Rockwool Block

**DOI:** 10.3390/plants14091378

**Published:** 2025-05-01

**Authors:** Jong Hyang Bae, Baul Ko

**Affiliations:** 1 Division of Horticulture Industry, Wonkwang University, Iksan 54538, Republic of Korea; bae@wku.ac.kr; 2 Institute of Life Science and Natural Resources, Wonkwang University, Iksan 54538, Republic of Korea; 3 Institute of Plant Breeding Research, Wonkwang University, Iksan 54538, Republic of Korea

**Keywords:** dry weight, leaf area, LED light, net assimilation rate, relative growth rate

## Abstract

This study was conducted to investigate the effects of different levels of photosynthetic photon flux density on the growth of paprika seedlings cultivated in rockwool blocks. The seedling height and internode length were significantly shorter in LED light treatments than in sunlight, and there was no difference among the PPFDs of LED light. On the other hand, leaf number and area of seedlings were significantly higher and wider, respectively, in sunlight than in 150 μmol·m^−2^·s^−1^ treatments. The influence levels on the dry weight were 20% lighter than those on the fresh weight. The higher the PPFD of LED light, the better the seedling quality. The leaf area and dry weight of seedlings in 200 μmol·m^−2^·s^−1^ treatments were 582 cm^2^/plant and 2.01 g/plant, respectively, higher by 35% and 70%, respectively, than in 100 μmol·m^−2^·s^−1^ treatments. The leaf area (Y_1_) had a significant dependence on PPFD (x), as Y_1_ = 75.3x + 368 (R^2^ = 0.9307 **). Also, the dry weight (Y_2_) of the seedlings showed a linear regression equation, as Y_2_ = 0.415x + 0.811 (R^2^ = 0.9674 **). The chlorophyll content based on the SPAD value significantly increased as the light intensity increased to 50 μmol·m^−2^·s^−1^. When the results were synthesized, the seedling quality was lower in the natural light conditions than in the 150 μmol·m^−2^·s^−1^ treatments.

## 1. Introduction

Paprika is one of the most representative high-value-added crops among greenhouse vegetables [1], and is regarded as a promising export crop. Since its introduction to Korea in 1994, its cultivation area has rapidly expanded to 734 ha, with a production volume of 77,880 tons in 2020 [2]. Fruit vegetables such as paprika are influenced by various factors, including light, temperature, humidity, and CO_2_, which affect their growth and yield after transplanting [3]. In particular, seedling quality is a crucial factor for post-transplant growth, as high-quality seedlings significantly influence plant growth, yield, and fruit quality after transplanting [4,5]. In Korea, paprika is cultivated entirely using hydroponic systems. Various studies have been conducted to improve seedling quality for hydroponic cultivation, including research on bending methods when trans-planting rock wool blocks [6,7], determining the optimal nursery period and rock wool block size [8,9], identifying suitable environmental conditions for seedling management [10], and utilizing artificial lighting to optimize light conditions in enclosed seedling production systems [11,12]. Light conditions, in particular, are essential for plant growth [13] and impact photosynthesis, the vegetative growth of plant organs, yield, and fruit quality [14,15]. Although paprika has a relatively low light requirement [7], with a light saturation point of 30–40 Klx, insufficient light intensity can limit photosynthesis and negatively affect plant growth [16]. Studies have shown that using fluorescent or LED lights during seedling production can shorten the initial harvest period by one week compared to natural light, thereby increasing early yield [17]. Additionally, when cultivating seedlings under LED light, a red-to-blue light ratio of 8:2 resulted in the highest growth rates. Moreover, using a closed-type nursery system with fluorescent lighting can shorten the seedling period compared to greenhouse cultivation [11]. Despite these studies proposing optimal light conditions to enhance seedling quality, their application in commercial cultivation remains limited. This study aims to investigate the growth characteristics of paprika seedlings cultivated on rock wool blocks under different photosynthetic photon flux densities (PPFD) using LED lighting. The findings will serve as fundamental data for practical applications in hydroponic paprika seedling production systems.

## 2. Results and Discussion

### 2.1. Growth Characteristics of Paprika Seedlings Under Different Photosynthetic Photon Flux Densities (PPFDs)

#### 2.1.1. Quality of Paprika Seedlings

The growth characteristics of paprika seedlings under different LED light intensities are shown in Table 1. The plant height was tallest under natural light at 16.1 cm, whereas the LED treatments ranged between 11.9 and 12.7 cm, showing no significant differences among the LED-treated groups. The stem diameter was thickest under natural light at 5.52 mm, while in the LED treatments, it increased with light intensity: 4.60 mm at 100 μmol·m^−2^·s^−1^, 4.89 mm at 150 μmol·m^−2^·s^−1^, and 5.26 mm at 200 μmol·m^−2^·s^−1^. The number of leaves was 13.6 under natural light, which was higher than that of the 100 μmol·m^−2^·s^−1^ LED treatment (12.7), but significantly lower than that of the 150 μmol·m^−2^·s^−1^ (14.7) and 200 μmol·m^−2^·s^−1^ treatments (15.2). The leaf area followed a similar trend, with 493 cm^2^/plant under natural light, which was higher than the 100 μmol·m^−2^·s^−1^ treatment (431 cm^2^/plant), but significantly lower than the other LED treatments, which ranged from 542 to 582 cm^2^/plant.

**Figure 1:**
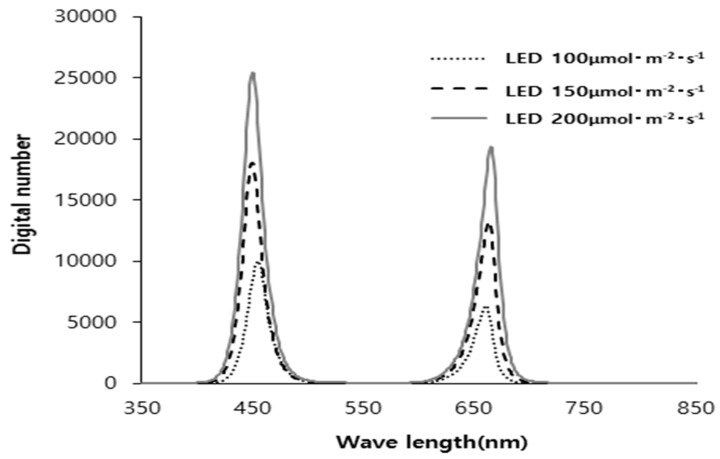
Spectral distribution by intensities (PPFDs) of LED light (red–blue = 1:1 ratio).

The shorter plant height under all LED treatments compared to natural light is consistent with previous studies reporting that shoot elongation is more pronounced under natural light than LED lighting [18]. Notably, in the 150–200 μmol·m^−2^·s^−1^ LED treatments, the number of leaves exceeded that of the natural light group. Given the plant height, this suggests that the internode length differences between natural light and LED treatments were greater than the differences in the plant height itself. This phenomenon can be attributed to the light quality of the LED sources used in this study, which included blue and red wavelengths that influence plant growth (Figure 1). These wavelengths interact with photoreceptors such as cryptochromes and phytochromes, which inhibit excessive stem elongation [19]. Specifically, cryptochromes responding to blue light (440 nm) suppress the synthesis of gibberellin (GA), thereby affecting hypocotyl elongation [20]. Furthermore, while LED treatments provide stable light intensity, the control group, relying solely on natural light, experienced fluctuations in light reception, leading to inconsistent growth, including potential etiolation [11].

The chlorophyll content, measured using the SPAD values, was 51.4 under natural light, 45.8 under 100 μmol·m^−2^·s^−1^, 50.0 under 150 μmol·m^−2^·s^−1^, and 55.1 under 200 μmol·m^−2^·s^−1^, showing a clear increase of approximately 5 SPAD units with every 50 μmol·m^−2^·s^−1^ increment in light intensity. This result is consistent with previous reports indicating that higher light intensities enhance chlorophyll content and photosynthetic rates [21,22]. These findings suggest that under the tested photosynthetic photon flux density (PPFD) conditions, LED intensities of 200 μmol·m^−2^·s^−1^ or higher are optimal for paprika seedling growth, as they promote shoot and leaf development while securing sufficient leaf area for photosynthesis after transplanting. Additionally, LED lighting can serve as an effective supplemental light source when utilizing natural light.

#### 2.1.2. Fresh and Dry Weights of Paprika Seedlings Under Different Light Intensities

The fresh and dry weights of paprika seedlings under different light intensities are presented in Table 2. The fresh weight was 20.2 g under natural light, while under LED treatments, it increased with light intensity: 13.9 g at 100 μmol·m^−2^·s^−1^, 19.2 g at 150 μmol·m^−2^·s^−1^, and 21.4 g at 200 μmol·m^−2^·s^−1^, showing no significant difference between the 200 μmol·m^−2^·s^−1^ LED treatment and natural light. However, the dry weight was significantly higher under natural light compared to the 200 μmol·m^−2^·s^−1^ LED treatment. Consequently, the dry matter ratio in the natural light group was 12.0, which was noticeably higher than the 8.6–9.4 range observed in LED-treated plants.

This trend may be attributed to the effects of blue light-responsive phytochromes, which contribute to leaf expansion, and cryptochromes, which suppress stem elongation, leading to differences in dry matter distribution among plant organs [23,24].

#### 2.1.3. Changes in Plant Height(A) and No. of Node(B) Under Different Light Intensities

**Figure 2:**
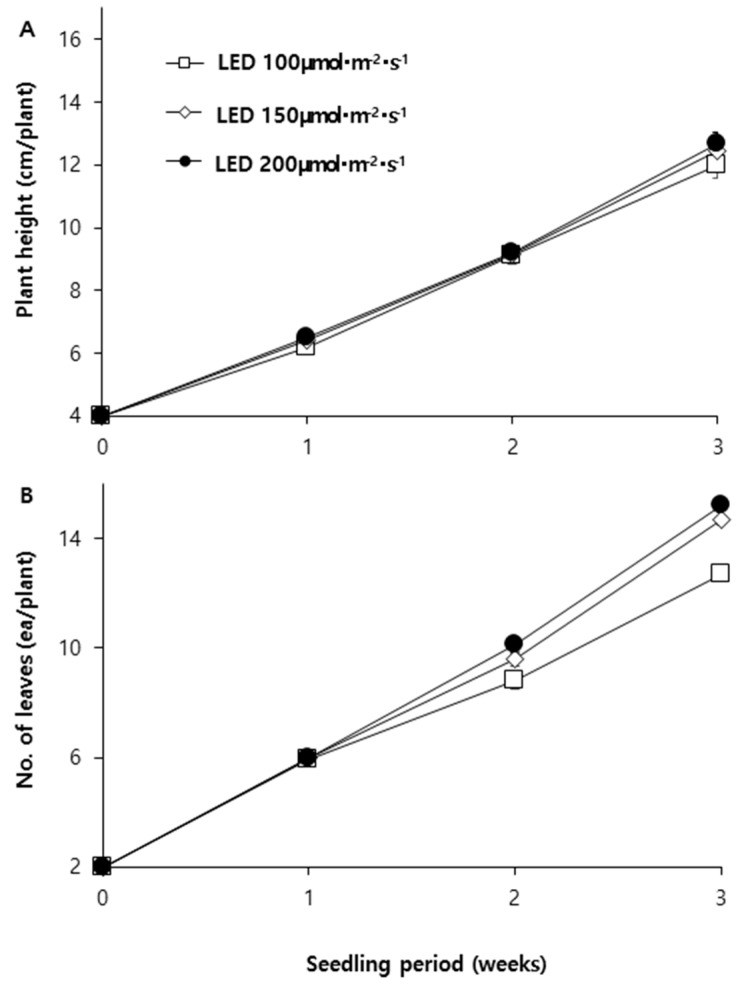
Changes in plant height (**A**) and leaf number (**B**) of paprika seedlings. Vertical bars represent the standard error of the mean (*n* = 10).

The changes in plant height and leaf number after transplanting are illustrated in Figure 2. Plant height showed no significant differences between treatments at any growth stage, maintaining a nearly identical growth pattern. However, the number of leaves exhibited differences from the second week after transplanting, showing a positive correlation with light intensity. In particular, from the second week, leaf expansion in the 100 μmol·m^−2^·s^−1^ LED treatment was significantly slower compared to the 150 μmol·m^−2^·s^−1^ or higher treatments [25].

#### 2.1.4. Fresh and Dry Weights of Paprika Seedlings

The relative growth rate, net assimilation rate, and leaf area ratio of paprika seedlings cultivated for three weeks under different light intensities are shown in Table 3. The relative growth rate was highest under natural light and increased with higher light intensity in LED treatments. The leaf area ratio exhibited the opposite trend, with the highest value (202.9) under natural light, and decreasing with increasing light intensity in LED treatments. The net assimilation rate, which represents the dry matter production ability per unit of leaf area, followed a similar trend to the relative growth rate, being highest under natural light and increasing with higher LED intensities. Specifically, the net assimilation rate was 0.294 under natural light, which was 1.5–2.3 times higher than in LED treatments, indicating higher photosynthetic efficiency in natural light conditions.

However, considering that the leaf area was larger under LED treatments than under natural light, the dry matter production per unit of plant volume is expected to be higher in LED-treated seedlings. Additionally, given that the experiment was conducted in June, a period with high solar radiation, controlling the solar radiation levels was challenging. This suggests that during cloudy seasons or winter, when light availability is unstable, LED lighting could serve as an effective supplemental light source for seedling production [26].

#### 2.1.5. Regression Analysis of Light Intensity and Seedling Growth Parameters

**Figure 3:**
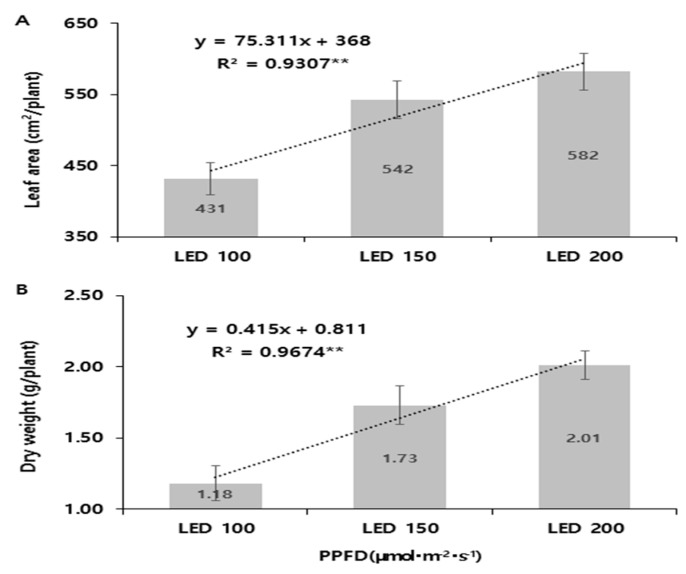
Relationship between intensities of LED light (red–blue = 1:1 ratio) and leaf area (**A**) and dry weight (**B**) of paprika seedlings. Vertical bars represent the standard error of the mean (*n* = 10).

The regression analysis between light intensity (X) and paprika seedling leaf area (Y_1_) and dry weight (Y_2_) is shown in Figure 3. The relationships were found to be linear, with the following equations:

- Leaf area (Y_1_) = 75.311X + 368 (R^2^ = 0.937) **.
- Dry weight (Y_2_) = 0.415X + 0.811 (R^2^ = 0.9674) **.

These results indicate a strong linear correlation between light intensity and seedling growth parameters, suggesting that higher light intensity contributes to increased leaf expansion and dry matter accumulation in paprika seedlings.

### 2.2. Irrigation Cycle Under Different Photosynthetic Photon Flux Densities (PPFDs)

#### 2.2.1. Irrigation Characteristics of Paprika Seedlings

**Figure 4:**
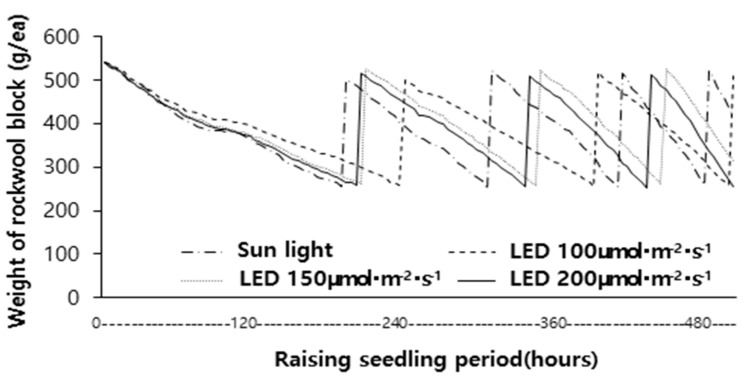
Changes in weight of rockwool block with paprika seedlings.

The water retention characteristics of rock wool blocks for paprika seedlings under different light intensities are shown in Figure 4 and Table 4. The total number of irrigation events was five under natural light, whereas it was three under 100 μmol·m^−2^·s^−1^, and four under both 150 μmol·m^−2^·s^−1^ and 200 μmol·m^−2^·s^−1^. This indicates that the number of irrigation events under natural light was 1–2 times higher than under LED treatments.

The average water retention time per irrigation event was 122 h under natural light, while under LED treatments, it was 180 h at 100 μmol·m^−2^·s^−1^, 153 h at 150 μmol·m^−2^·s^−1^, and 147 h at 200 μmol·m^−2^·s^−1^. The higher number of irrigation events and shorter water retention time under natural light may be attributed to limited ventilation due to shading screens in LED treatments, which led to higher relative humidity and a lower vapor pressure deficit compared to natural light conditions.

#### 2.2.2. Effects of Light Intensity on Internal Temperature of Shading Screens

**Figure 5:**
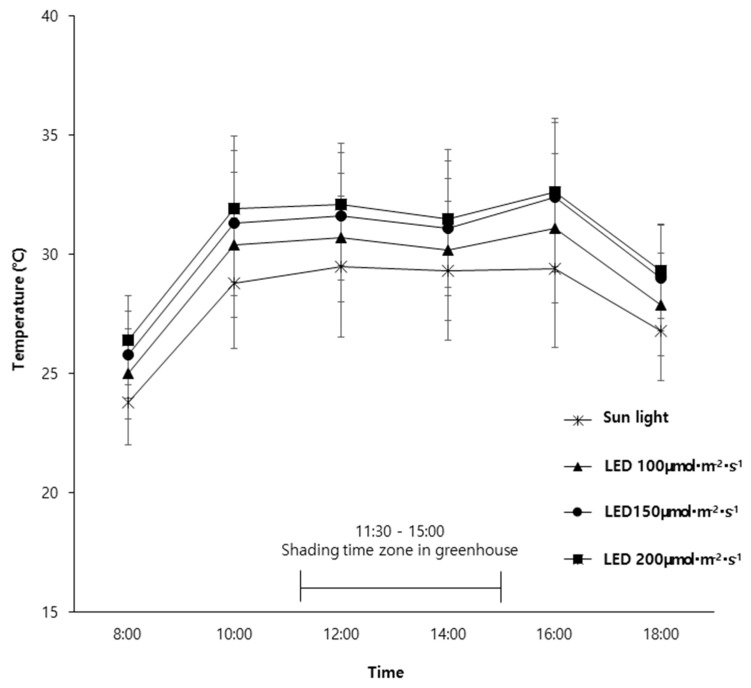
Difference in daily mean temperature in shade net. Vertical bar represents the standard deviation of the mean.

Measurements of the internal temperature within the shading screen under different light intensities (Figure 5) revealed that under natural light, the average temperature was 28 °C, whereas under LED treatments, the average temperatures were 29.2 °C at 100 μmol·m^−2^·s^−1^, 30.2 °C at 150 μmol·m^−2^·s^−1^, and 30.7 °C at 200 μmol·m^−2^·s^−1^. These results indicate that LED-treated environments were 1.2–2.7 °C higher than natural light conditions, with temperature increasing by approximately 0.5–1.5 °C as LED intensity increased.

This trend is likely due to differences in heat dissipation based on light intensity. While LEDs are known for their high energy efficiency, they still generate heat, and the amount of heat produced increases as light intensity rises [19]. These results align with previous findings where leaf temperature was higher under LED supplementary lighting compared to non-illuminated (natural light) conditions [18].

Considering the water loss from rock wool blocks and the internal temperature of the shading screens under different LED intensities, the shorter average water retention time under 150 μmol·m^−2^·s^−1^ and 200 μmol·m^−2^·s^−1^ compared to 100 μmol·m^−2^·s^−1^ suggests that differences in the internal temperature affected the evapotranspiration rates. This cumulative effect over the experimental period may have contributed to growth differences among LED treatments. Therefore, further detailed research under controlled temperature conditions is required to better understand these effects.

#### 2.2.3. Water Retention Duration per Irrigation Event

**Figure 6:**
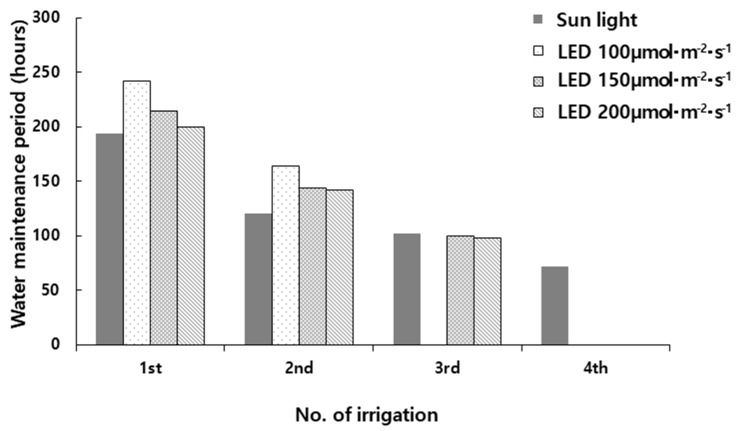
Changes in water maintenance period of rockwool block with paprika seedlings.

As shown in Figure 6, the water retention duration per irrigation event varied across treatments:

- First irrigation event: 194 h under natural light, 242 h under 100 μmol·m^−2^·s^−1^, 214 h under 150 μmol·m^−2^·s^−1^, and 200 h under 200 μmol·m^−2^·s^−1^;
- Second irrigation event: 120 h under natural light, 164 h under 100 μmol·m^−2^·s^−1^, 144 h under 150 μmol·m^−2^·s^−1^, and 142 h under 200 μmol·m^−2^·s^−1^;
- Third irrigation event: 102 h under natural light, 100 h under 150 μmol·m^−2^·s^−1^, and 98 h under 200 μmol·m^−2^·s^−1^.

From the third irrigation event onward, the water retention duration under LED treatments was shorter than that under natural light. This trend may be due to increased water demand and transpiration rates associated with enhanced plant growth under higher LED intensities.

#### 2.2.4. Optimal LED Intensity for Efficient Irrigation Management

Based on these results, the 100 μmol·m^−2^·s^−1^ LED treatment exhibited a significantly lower leaf area and dry weight, indicating poorer seedling quality when using LED as the primary light source for paprika seedling production.

Within the experimental design range, although the total number of irrigation events was the same between 150 μmol·m^−2^·s^−1^ and 200 μmol·m^−2^·s^−1^, the 200 μmol·m^−2^·s^−1^ treatment resulted in greater plant growth. Thus, considering the cost–benefit ratio, 200 μmol·m^−2^·s^−1^ appears to be the most efficient light intensity for paprika seedling production using LED lighting.

## 3. Materials and Methods

### 3.1. Seedling Method

This study was conducted in a plastic film greenhouse located at the Department of Horticultural Industry, Wonkwang University, using the paprika cultivar Scirocco (Enza Zaden Co., Enkhuizen, The Netherlands). Seeds were sown in 240-cell rock wool plugs (20 × 27 mm, Grodan Co., Roermond, The Netherlands) and germinated in a controlled growth chamber at 25 ± 1 °C temperature and 90% relative humidity. After germination, the seedlings were transferred to natural light conditions for management. When the seedlings had developed two true leaves, they were transplanted into rock wool blocks (10 × 10 × 6.5 cm, Grodan Co., The Netherlands) using a U-shaped bending method at a rate of 10–20 plants per treatment [6].

The nutrient and water supplies were provided using the standard paprika nutrient solution recommended by the Rural Development Administration (RDA), diluted to a pH of 5.8 and EC of 1.8 dS·m^−1^. Irrigation was supplied via a sub-irrigation system, maintaining a 50% moisture level in the rock wool blocks [27]. The seedling period was set at three weeks (21 days) after transplantation into rock wool blocks. The experiment was conducted in June to accommodate the summer cropping season.

### 3.2. Measurement Items and Methods

#### 3.2.1. Growth Measurements of Seedlings

At the end of the seedling period, destructive sampling was performed, and the shoot was examined after severing the hypocotyl base from the rock wool block. The growth parameters measured included plant height, stem diameter, number of leaves, leaf area, fresh weight, and dry weight.

- Plant height: Measured from the base to the shoot apex;
- Stem diameter: Measured from 1 cm above the cotyledonary node using a vernier caliper (500-182, Mitutoyo Co., Kawasaki, Japan);
- Leaf number: Counted after separating the leaves from the stem;
- Leaf area: Measured using a leaf area meter (LI-3100, LI-COR Co., Lincoln, NE, USA);
- Fresh weight and dry weight: Measured using an electronic balance, and dry weight was determined after drying the samples at 60 °C for over 72 h in a drying oven.

#### 3.2.2. Growth Analysis of Seedlings

For growth analysis, destructive measurements were taken both before and after transplantation, and the following growth parameters were calculated based on the Rural Development Administration’s [28] Agricultural Science and Technology Research Standards:

1. **Relative Growth Rate (RGR)**

(1) $$\frac{\log_{e}DW_{2}-\log_{2}DW_{2}}{T_{2}-T_{1}}$$

2. **Net Assimilation Rate (NAR)**

(2) $$\frac{\left(DW_{2}-DW_{1}\right)\times \left(log_{e}DW_{2}-\log_{e}DW_{1}\right)}{\left(T_{2}-T_{1}\right)\times \left(L_{2}-L_{1}\right)}\times D\;\mathrm{NAR}\;=\;(T_{2}-T_{1})\;\times \;(L_{2}-L_{1})({\mathrm{DW}}_{2}-{\mathrm{DW}}_{1})\times ({\mathrm{logDW}}_{2}-{\mathrm{logDW}}_{1})\times D$$

3. **Leaf Area Ratio (LAR)**

(3) $$\frac{L}{DW}$$

4. **Dry Matter Ratio (Dry Matter Content, DM%)**

(4) $$\left(\frac{DW}{FW}\right)\times 100$$

where

- L = leaf area per plant (cm^2^);
- DW = dry weight per plant (g);
- FW = fresh weight per plant (g);
- DW_1_, DW_2_ = dry weight at initial and final measurement (g);
- T_1_, T_2_ = time interval between measurements (days);
- L_1_, L_2_ = leaf area at initial and final measurement (cm^2^);
- D = planting density in the greenhouse.

### 3.3. Experimental Treatments

**Figure 7:**
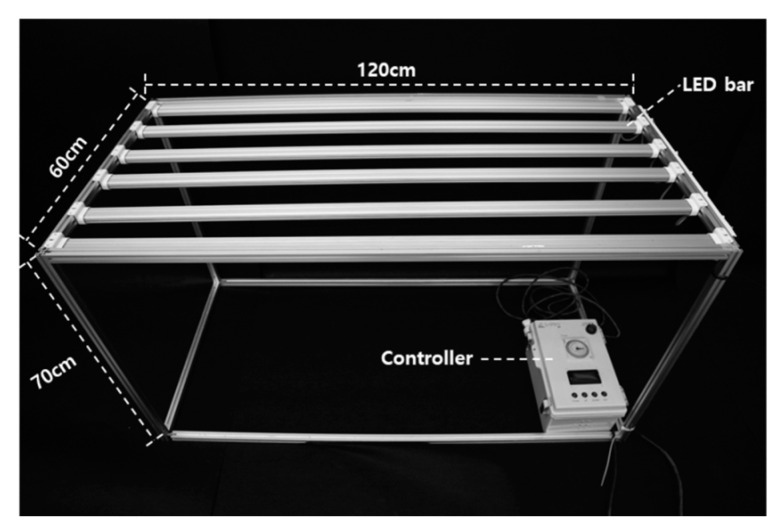
Device for testing the effects of various intensities of LED light on paprika seedlings in this study.

The experiment was conducted in a plastic film greenhouse without environmental control. The light source used was a custom-built LED system (Figure 7) installed on a 120 × 60 × 70 cm frame, equipped with LEDs emitting wavelengths that affect plant growth at a red–blue ratio of 1:1, providing a maximum light intensity of 250 μmol·m^−2^·s^−1^.

The experimental treatments were based on different photosynthetic photon flux density (PPFD) levels at the plant’s shoot apex immediately after transplanting into rock wool blocks:

- 100 μmol m^−2^·s^−1^;
- 150 μmol m^−2^·s^−1^;
- 200 μmol m^−2^·s^−1^.

Additionally, a natural light treatment was included to simulate summer seedling production in a commercial setting. The light spectrum for each treatment is shown in Figure 7.

To minimize the influence of natural light, a 90% blackout shading screen was used, ensuring internal air circulation. A dry-bulb thermometer was installed inside the shading chamber to monitor the temperature variations under different light intensities.

### 3.4. Statistical Analysis

The collected data were analyzed using SPSS statistical software (Version 12.0, IBM Co., Armonk, NY, USA) and Microsoft Excel (MS Office 2016, Microsoft Co., Redmond, WA, USA). Statistical significance among treatments was determined using Duncan’s Multiple Range Test (DMRT) at a 95% confidence level.

## Figures and Tables

**Table 1:** Growth characteristics of paprika seedlings.

Treatment (μmol·m^−2^·s^−1^)	Plant Height (cm/plant)	Stem Diameter (mm/plant)	Leaf Number (no./plant)	Leaf Area (cm^2^/plant)	Chlorophyll Content (SPAD)
Sunlight	16.1 a ^z^	5.52 a	13.6 c	493 b	51.4 b
100	11.9 b	4.60 d	12.7 d	431 c	45.8 c
150	12.5 b	4.89 c	14.7 b	542 a	50.0 b
200	12.7 b	5.26 b	15.2 a	582 a	55.1 a

^z^ Mean separation within columns by Duncan’s multiple range test at *p* = 0.05.

**Table 2:** Fresh and dry weights and dry masses of paprika seedlings.

Treatment (μmol·m^−2^·s^−1^)	Fresh Weight (g/plant, A)	Dry Weight (g/plant, B)	Dry Mass [%, (B/A)*100]
Sunlight	20.2 a ^z^	2.43 a	12.0 a
100	13.9 c	1.18 d	8.6 d
150	19.2 b	1.73 c	9.0 c
200	21.4 a	2.01 b	9.4 b

^z^ Mean separation within columns by Duncan’s multiple range test at *p* = 0.05.

**Table 3:** Relative growth rate (RGR), net assimilation rate (NAR), and leaf area ratio (LAR) of paprika seedlings.

Treatment (μmol·m^−2^·s^−1^)	RGR (g g^−1^ d^−1^)	NAR (g cm^2^ d^−1^)	LAR (cm^2^ g^−1^)
Sunlight	0.169 a ^z^	0.294 a	202.9 d
100	0.134 d	0.126 d	367.1 a
150	0.153 c	0.168 c	315.5 b
200	0.160 b	0.193 b	289.5 c

^z^ Mean separation within columns by Duncan’s multiple range test at *p* = 0.05.

**Table 4:** Irrigation levels supplied for producing paprika seedlings for 3 weeks under various intensities of LED light (red–blue = 1:1 ratio).

Treatment (μmol·m^−2^·s^−1^)	Total Irrigation Number (Season)	Average of Water Maintenance Periods (Hours/Time)
Sunlight	5	122
100	3	180
150	4	153
200	4	147

## Data Availability

All data are available within the paper.
